# Quantifying and Mapping Global Data Poverty

**DOI:** 10.1371/journal.pone.0142076

**Published:** 2015-11-11

**Authors:** Mathias Leidig, Richard M. Teeuw

**Affiliations:** School of Earth and Environmental Sciences, University of Portsmouth, Portsmouth, Hampshire, United Kingdom; East China University of Science and Technology, CHINA

## Abstract

Digital information technologies, such as the Internet, mobile phones and social media, provide vast amounts of data for decision-making and resource management. However, access to these technologies, as well as their associated software and training materials, is not evenly distributed: since the 1990s there has been concern about a "Digital Divide" between the data-rich and the data-poor. We present an innovative metric for evaluating international variations in access to digital data: the Data Poverty Index (DPI). The DPI is based on Internet speeds, numbers of computer owners and Internet users, mobile phone ownership and network coverage, as well as provision of higher education. The datasets used to produce the DPI are provided annually for almost all the countries of the world and can be freely downloaded. The index that we present in this ‘proof of concept’ study is the first to quantify and visualise the problem of global data poverty, using the most recent datasets, for 2013. The effects of severe data poverty, particularly limited access to geoinformatic data, free software and online training materials, are discussed in the context of sustainable development and disaster risk reduction. The DPI highlights countries where support is needed for improving access to the Internet and for the provision of training in geoinfomatics. We conclude that the DPI is of value as a potential metric for monitoring the Sustainable Development Goals of the Sendai Framework for Disaster Risk Reduction.

## Introduction

It is clear that socio-economic poverty results in greater vulnerability to the impacts of hazards and reduced resilience when disasters hit [1, 2]. According to Mutter [3] “people in countries ranked among the lowest 20 percent in the Human Development Index are 10 to 1,000 times more likely to die in a natural disaster than people from countries in the top 20 percent”.

Less widely recognised is the importance of information for sustainable development and disaster risk reduction, particularly the impacts of data poverty. As noted by the International Federation of Red Cross & Red Crescent Societies (IFRC, 2005, p.12) [4]: “Information is also a vital form of aid in itself. People need information as much as water, food, medicine or shelter. Information can save lives, livelihoods and resources. It may be the only form of disaster preparedness that the most vulnerable can afford. And yet it is very much neglected.“

In the 1990s the term “digital divide” was introduced to describe the gap between those who have—and those who do not have—access to computers, the Internet and the corresponding computer literacy [5, 6]. Other terms used to describe that disparity include “information inequality” or “information gap” [7]. The term “information poverty” was used by Baban et al. [8], to describe a lack of effective and reliable information for the decision-making of land-use planners in the Caribbean region. In this article we examine the Digital Divide concept in the context of our increasingly digital world, in which a vast amount of data is now provided via the Internet and with over 75% of the world population owning a mobile phone [9]. Data poverty thus reflects the ability to access online data, information and resources such as teaching materials, as well as the capability to share data and information. We present a Data Poverty Index (DPI), with the resulting map of global variations in data poverty, and consider those variations in the context of sustainable development and disaster risk reduction.

Information technologies play key roles in sustainable development and disaster risk reduction, facilitating the generation and dissemination of knowledge. There are many types of geospatial data, such as satellite images, measurements used for weather forecasts and storm warnings, the Global Positioning System (GPS) co-ordinates of land tenure boundaries, and the digital elevation models that provide 3-D views of landscapes in virtual globes, such as Google Earth. Geoinformatics is used here as a catch-all term, for geospatial data and the technologies used to collect it, as well as the software for processing it. Many sets of free software and data are available, mostly via internet downloads, that could be used for activities that assist sustainable development, such as analysis of national census data, mapping types of farmland, monitoring of urbanisation, or the preparation of preparedness maps for Disaster Risk Reduction [10]. Of particular use for guiding decision-makers, are digital datasets that can be analysed and processed by Geographical Information Systems (GIS). Those decision-makers can range from farmers in remote regions, through to executive officers of government.

Organisations dealing with sustainable development generally require easy-to-use and quick to implement indicators to quantify poverty. However, finding detailed metrics is challenging, due to a lack of resources, time and expertise [11]. There is a need for standardized indicators to evaluate the impacts of development programmes. Ideally, such indicators follow the SMART criteria: Specific, Measureable, Available cost-effectively, Relevant and Timely available [12, 13]. Traditional measurements require paper-based questionnaires that are slower to process and more costly to produce than digital surveys and metrics. The digital approach of the Data Poverty Index provides a rapid low-cost method for annual evaluations of global access to digital data, using national metrics that are currently freely available.

The United Nation’s 2005 Hyogo Framework proposed five actions:

1. making DRR a policy priority, with more community involvement;
2. more risk assessment and early warning systems;
3. improved education, information and public awareness;
4. reducing underlying risk factors;
5. better preparedness and effective response [14].

Geospatial data and geoinformatic technologies are of use for all of the Hyogo actions: reducing the data poverty of individuals and communities will reduce their vulnerability, making them better prepared for disasters and more resilient to their impacts, thus reducing disaster risk. The Sendai Framework for Disaster Risk Reduction (SFDRR) 2015–2030 indicates that exposure to disaster, of persons and assets (e.g. buildings, critical infrastructure), in all countries will increase in frequency and intensity [15]. The DPI is a possible metric to monitor the implementation of the SFDRR. It provides a rapid method for annual analysis, addressing the challenges that many developing countries face with regard to the adequate, timely and sustainable provision of data and information, through technology transfer and capacity building. The DPI directly links to the SFDRR disaster risk communication aspect, which aims to “Promote and enhance, through international cooperation, including technology transfer, access to and the sharing and use of non-sensitive data, information [..] communications and geospatial and space-based technologies and related service [..] strengthen the utilization of media, including social media, traditional media, big data and mobile phone networks, to support national measures for successful disaster risk communication” [15].

Freely available software can support the development of user-friendly systems and services for the exchange of data and information to assist sustainable development and disaster risk reduction at local, district, national, regional and global levels. Sustainable usage of free data and the free software for data processing is dependant on the provision of freely-available training and education, which is also evaluated in the Data Poverty Index [16–18].

Satellite imagery is widely accepted as the best source of data for mapping and monitoring earth-surface features, such as land cover types or hazardous terrain, particularly areas that are remote or inaccessible [19, 20]. Disaster response has become a prominent application domain of satellite imagery, as seen in disasters such as the Indian Ocean Tsunami (2004), the Haiti earthquake (2010), and Typhoon Hayan (2013). During the past decade, archives of satellite imagery with near-global coverage have become freely available via the Internet, such as the Global Digital Elevation Model (G-DEM), the USGS Landsat archive and the Sentinel Archive of the European Space Agency [21, 22]. These geospatial data archives enable the production of disaster preparedness maps that can highlight districts at risk of disaster and guide emergency planners.

Modern desktop and laptop computers are powerful enough to run software for geospatial analysis and map-making, using geoinformatic data, with much of the software and training material freely available via Internet downloads [10, 18]. However, access to the Internet varies considerably, both internationally and within individual countries. While the Internet offers huge possibilities for the global transfer of data and information, access to the Internet is not evenly distributed: that is a limitation on sustainable development that we examine here.

## Methodology

**Fig 1:**
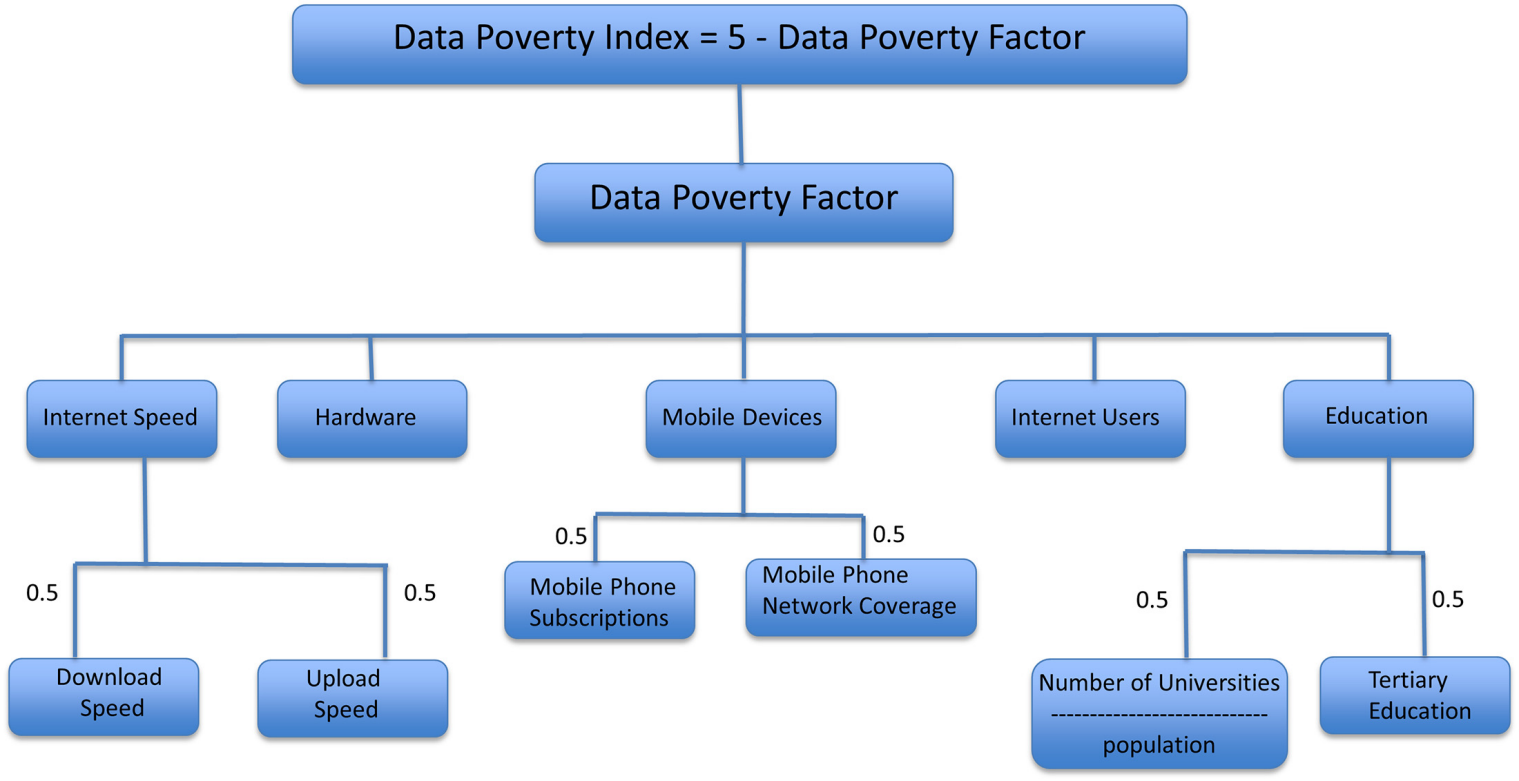
Data sets input to calculate the Data Poverty Index.

The input data for the Data Poverty Index is entirely derived from currently freely available sources. The majority of the input data sets were obtained from the World Bank website (http://data.worldbank.org/), which provides data that tends to be more up-to-date than data from the United Nations website (http://data.un.org). Data on the World Bank and UN website are typically updated yearly. Information for the Internet-speed comes from the Net-Index website (http://www.netindex.com/) to ensure governmental independent data. At the time when this research was carried out (September 2014), the Net-Index data set was accessible via the Internet and daily average Internet speeds could be downloaded for all countries from its archive. However, not all datasets are freely available on a regular basis; for instance, the netindex.com website data set has not been available since mid-2015, although an alternative source of Internet Speed data can now be found at: http://www.ookla.com. The data regarding mobile phone networks was obtained from the World Bank website, but originates from the International Telecommunication Union (ITU). The ITU data sets tend to only be freely available online for a limited amount of time. The most recently available datasets have been used to calculate, map and visualise global data poverty. Weblinks for data sources used to calculate the Data Poverty Index (DPI) factors are given in Table 1, with a graphic summary provided in Fig 1.

**Table 1:** Weblinks for data sources used for the DPI factors.

Factor	Year of the most recent data used	Data Source
		(all data downloaded on 05 September 2014)
**Internet Speed**		
Upload and Download Speed [kbps to Mbps]	2013	http://www.netindex.com/ *
**Hardware**		http://data.worldbank.org
Percent of households with a computer	2013	From WDI table 5.12: “The Information Society”; original source: ITU.
**Mobile Devices**		
Mobile Phone Subscriptions: *“Mobile cellular Subscriptions per 100 people (2013)”*	2012 and 2013	http://data.worldbank.org/
Mobile Network Coverage: *“Telephones quality; Population covered by mobile cellular network; % (2012)”*		From WDI table 5.11:“Power & communications”, original source: ITU. Missing data filled with data of same year, from http://data.un.org
**Internet Users**		http://data.worldbank.org/
Individuals using the Internet: % of population.	2013	From WDI table 5.12: “The Information Society”, original source: ITU. Missing data filled with data of same year, from http://data.un.org
**Education**		
Number of universities (2014)	2012 to 2014	http://whed.net missing data filled with data from 4icu.org;
Population (millions, 2013)		http://data.un.org;
People in tertiary education (2012, 2013)		http://data.worldbank.org/; WDI table 2.11: “Precipitation in Education (2013)”. Missing data filled with data from http://data.un.org “Gross enrolment ratio in tertiary education (2012)”

* Since mid-2015 the netindex.com website is no longer accessible; however an alternative source of Internet Speed data is: http://www.ookla.com.

The Data Poverty Index is derived from five factors (Fig 1), which are described below:

1. **Internet speed—**a reliable and fast Internet connection is needed to download data; to share and/or upload data; e.g. to view or contribute to social media and volunteered geographic information (VGI) initiatives, such as crowd-source mapping [23–25]. The download speeds used in this evaluation range from 0.55 Mbps to 69 Mbps. To obtain a fair comparison of the countries, a download speed of 10 Mbps or greater has been allocated the highest score (i.e., 1.0) in the Internet Speed/Download category. This equates to downloading a DVD (4.7 GB) in 60 minutes. The data download score classes are based on the authors’ experience with geoinformatic fieldwork, training and conferences in many countries, from Europe to Africa, Asia and the Caribbean, where slow (sometimes non-existent) download speeds severely limited the amounts of data, software or training materials that could be accessed for disaster risk reduction projects. That the thresholds for the Internet speeds are reasonable, is further illustrated by the equipment of a major UK Fire and Rescue Service (Hampshire: HFRS), which currently uses equipment that allows a transmit and receive data rate of 492Kbps. HFRS also uses the 2G (GPRS) to 3G (HSPA) wireless broadband standard. The 3G standard theoretically allow a download rate of 7.2 Mbps, but that is rarely met, in particular during an emergency response: 2–3 Mbps is typical. Faster wireless broadband, such as 4G (LTE), is currently not extensively used in disaster response situations, not least due to insufficient network coverage. Another major issue for emergency responders in the aftermath of a disaster is slower speed in mobile networks because of concurrent usage.
  Internet upload speed limits the dissemination and sharing of information and data. The maximum threshold for the upload speed was set to 1 Mbps. This is equivalent to 7.5 Mb per minute, which is the time required for the upload of two to three 12 mega-pixel digital pictures per minute. For the year examined in this study (2013), that could be achieved by the majority of countries (171 of 192). Access to the Internet and related technology can facilitate data management and dissemination including the use of social media and access to online VGI initiatives.
2. **Hardware**—the percentage of population possessing a computer indicates the level of technological understanding and the likely information and communication technology (ICT) training requirements for a given country or region. It also indicates the potential of a country’s population to access online maps, or to assist with the production of such maps, e.g. for disaster preparedness mapping.
3. **Mobile-Device availability—**this is based on mobile phone subscriptions per 100 people and mobile network coverage (percent of population covered by mobile cellular networks). This influences the potential of a country to get early warnings, for instance of adverse weather that might affect farmers or fisher folk [26]; it can also contribute to disaster response efforts, as in the aftermath of the Haiti earthquake [24, 25]. For countries that had data for mobile phone subscriptions, but no data for network coverage, we used this rule: if the percentage of subscriptions was larger than 100%, then it equated to a network coverage of 95%; otherwise the network coverage equalled the percentage of mobile phone subscriptions. The rationale being that, in the entire dataset, when the percentage of phone subscriptions was more than 100% (meaning that a number of people must have at least two mobile phones), then there was at least 96% network coverage. Countries having more than 100% in the mobile phone subscription category scored the full value of 1.0.
4. **Internet usage—**this is the percentage of individuals in a given country using the Internet. This indicates the proportion of a national population familiar with the Internet and how many people are likely to benefit from Internet-delivered resources.
5. **Education—**derived from the tertiary education enrolment ratio [27] and the quotient of the number of universities in a country, relative to the population of that country. This variable indicates the level of potential ‘computer literacy’ and hence provides an indication of the understanding of geoinformatic data and technologies, such as GPS or GIS, in a given country. The information about university provision was obtained from the World Higher Education Database [28] and the 4icu.org website [29]. To remove extreme values for small countries that have one university for relatively few inhabitants (e.g. San Marino) and to ensure a fair representation when comparing with other countries, the feature scaling was capped at 10, which results in the top-scoring countries having at least one university per 100,000 people. For the calculation of the Data Poverty Index, all input data was feature-scaled (0–1) to provide a comparable representation of the individual variables. The calculated Data Poverty Factor was subtracted from the maximal score of 5 to obtain a nominal range of values for the Data Poverty Index (low values for minor data poverty, high values for severe data poverty). When calculating the DPI, there is currently no evidence to support any one feature being weighted more highly than another, consequently no further scaling, weighting or ranking was applied to the Data Poverty Index variables. An issue that needs to be considered by further research is that the relative importance of a single variable might vary from application to application and between different disaster situations. For instance, the data requirements differ for long-term sustainable development versus those of rapid disaster responses. Internet speed and mobile phone usage are very important for search and rescue activities after earthquakes, but for sustainable development applications, the "education" or “hardware” variables, indicating ability to make use of digital information, might play a more significant role [30].

The allocation of weightings is an aspect of the DPI that requires more research: detailed analysis of optimal weightings is beyond the scope of this preliminary paper. However, to better understand the relationship of the factors used for the DPI calculation some statistics has been performed on the data from 152 countries (Table 2). The strong correlation between Hardware and Internet-users, indicates (not surprisingly) that PCs are used to access the Internet. For all other combinations there are weak to moderate, but significant, correlations.

**Table 2:** Statistical assessment of the DPI factors.

Factor combi-nation	Pearson Coefficient R	Determination Coefficient R^2^	t-value	p-value	t_crit_ for p = 0,05 (one tailed)	t > t_crit_	critical value for R (p = 0,05)
1/2	0,58	0,33	8,66	3,56E-15	1,66	True	0,13
1/3	0,60	0,36	9,16	1,83E-16	1,66	Ture	0,13
1/4	0,31	0,10	4,03	4,39E-05	1,66	True	0,13
1/5	0,55	0,31	8,15	6,62E-14	1,66	True	0,13
2/3	0,94	0,89	34,14	6,38E-73	1,66	True	0,13
2/4	0,52	0,27	7,40	4,40E-12	1,66	True	0,13
2/5	0,70	0,49	12,05	4,01E-24	1,66	True	0,13
3/4	0,52	0,27	7,45	3,42E-12	1,66	True	0,13
3/5	0,70	0,49	12,06	3,79E-24	1,66	True	0,13
4/5	0,47	0,22	6,54	4,61E-10	1,66	True	0,13

Factor 1: Internet Speed; Factor 2: Internet Users; Factor 3: Hardware; Factor 4: Mobile Devices; Factor 5: Education. Remark: the number of samples (countries with complete datasets) is 152.

The DPI methodology provides data that is detailed enough to allow comparison between countries. Nevertheless it could be modified for more detailed analysis, such as comparisons between the rural districts and cities of a given country. That might enable some useful analysis of data poverty variations between, for instance, lowland districts and mountainous districts, or coastal districts and remote interior districts. Volunteered Geographic Information (VGI) and similar approaches to crowd-source mapping appear to be suitable for more detailed (e.g. district level) analysis. An example of how that could be done was recently provided by Wesolowski et al. [31] for mobile network data, related to social connectivity and the spread of *Ebola* in Africa.

## Results

**Fig 2:**
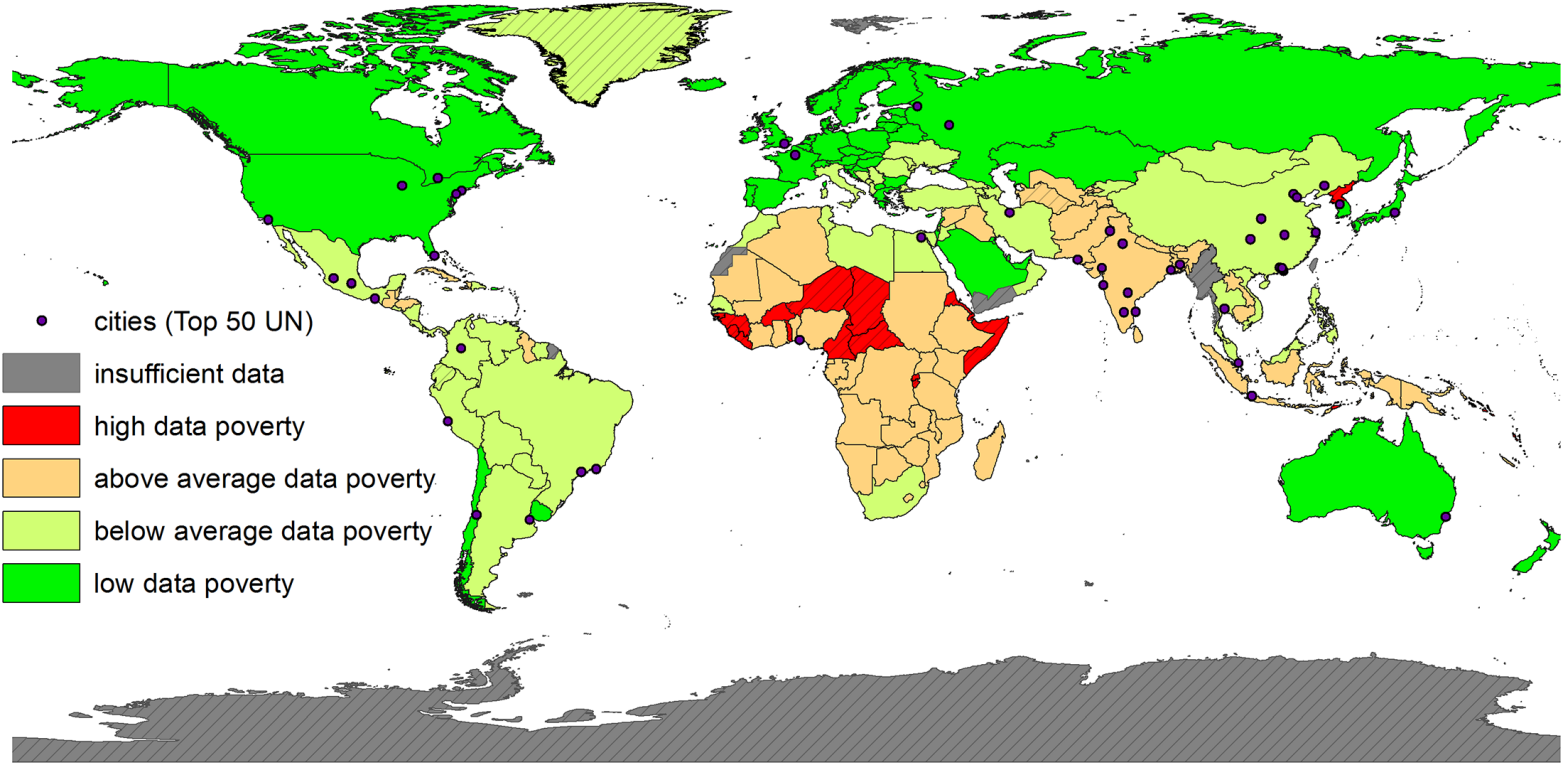
Map showing global Data Poverty for 2013, by nation states. The locations of the 50 most populous cities are also shown. The base map (world borders) was obtained from http://diva-gis.org/data.

The Data-Poverty Index provides results for 189 of the 214 countries listed by the World Bank. Of the countries that had data available for analysis, 152 have a complete data set; 37 had a near-complete data set, of which 11 have half of a variable-pair missing, usually the data about tertiary education or university provision; 18 are missing one variable, mostly the Internet download/upload speed; and 8 are lacking data for ‘one and a half’ variables. In the resulting global Data Poverty map (Fig 2), countries with incomplete data are indicated, along with countries for which there was insufficient relevant data. Table 3 indicates results for selected countries, including the highest and lowest scores.

**Table 3:** Example scores of the Data Poverty Index (DPI) and relationships to the World Bank income classification.

**Top Scores**		**Bottom Scores**	
***Country***	***Score (max*. *5)***	***Country***	***Score (max*. *5)***
1. Iceland	0.17	83. China*	2.05
2. Norway	0.36	109. Indonesia	2.75
3. Finland	0.39	114. Nigeria	2.88
4. Estonia	0.51	129. India	3.16
5. Denmark	0.52	142. Benin	3.49
8. U.S.A	0.55	148. Congo, Dem.	3.67
17. United Kingdom	0.71	149. Malawi	3.72
21. Germany	0.76	150. Yemen	3.78
23. Japan	0.77	151. Myanmar	3.95
39. Russia	1.04	152. Burkina Faso	4.04
**2014 World Bank income classification**		**Data Poverty Index Range**	
Low-income countries		4.04–2.62	
Lower-middle income countries		3.78–1.41	
Upper-middle income countries		3.32–0.97	
High-income countries		1.53–0.17	

Scores: < 1.21, high data poverty; 1.21–2.42, above average data poverty; 2.42–3.62, below average data poverty; > 3.62, low data poverty. Remark: Only countries with a complete dataset have been considered.

* China Mainland, excluding Macao and Hong Kong.

The number of countries in each class differs. Countries in the World Bank’s high income class have the most complete sets of data for the DPI calculation. Of the countries with a complete dataset: 54 are high-income, 41 are upper-middle-income, 38 are lower-middle-income and 19 are low-income. For low and lower-middle income countries, it would be particularly beneficial if the World Bank and UN could encourage them to provide the required data for calculating the DPI in future, so that their ICT development can be monitored.

**Fig 3:**
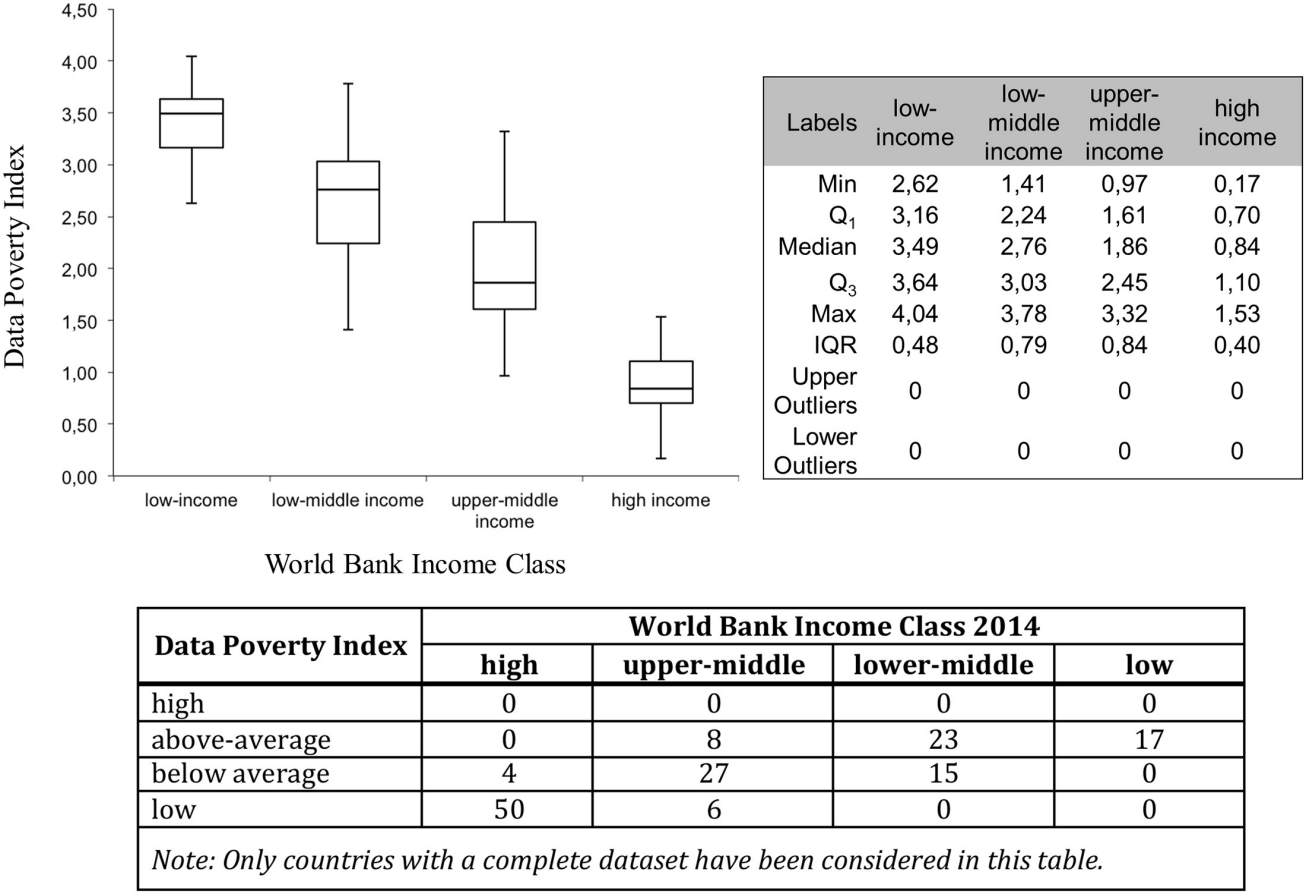
The Data Poverty Index in relation to World Banks Income classification. The ends of the whisker are set at 1.5*Interquartile Range (IQR) above the third quartile (Q3) and 1.5*IQR below the first quartile (Q1).

Regarding the Data Poverty map (Fig 2), African countries tend to score below average, as does most of South-East Asia. Conversely, South and Middle America, as well as East Asia, generally score above average. North America, Europe and Australia, as well as Russia and parts of Arabia, have minimal data poverty. Individual scores, for the top-scoring and bottom-scoring countries, are given in Table 3. Summary scores for the Data Poverty Index, relative to the income classification of the World Bank, are also shown in Table 3. Analysing the Data Poverty Index in relation to World Bank’s income classes with a box-whisker plot (Fig 3) indicates that there are no outliers among the classes.

**Fig 4:**
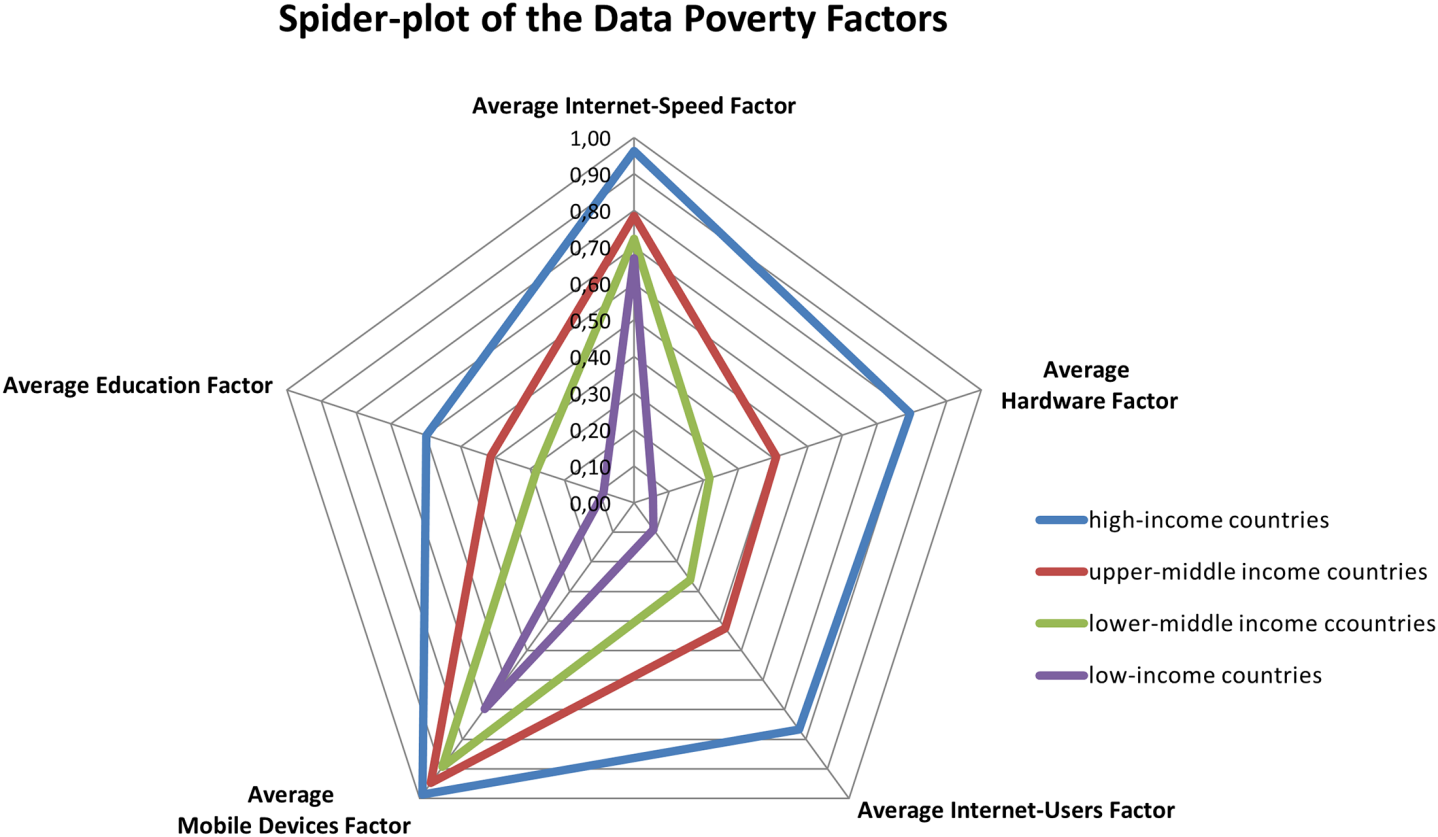
Spider plot indicating the average contribution of each factor to the DPI score of the corresponding World Bank income class.

The median in the Box-Whisker plots for low and low-middle income countries is towards the higher end of the DPI (high data poverty) while for upper-middle income and high-income countries it is at the lower end (low data poverty). There is a transition among the DPI scores of the lower-middle income class and upper-middle income class which indicates that data poverty can be addressed even with limited resources. How much each factor contributes, on average, to the DPI score of the corresponding World Bank income class is shown in Fig 4 (with corresponding values in Table 4).

**Table 4:** Overview of the average DPI factor scores compared to the World Bank income classification.

World Bank Income Class	Average Internet-Speed Factor	Average Hard- ware Factor	Average Internet-Users Factor	Average Mobile Devices Factor	Average Edu-cation Factor	Average DPI Factor
*high*	0,96	0,79	0,77	0,99	0,60	***0*,*89***
*upper-middle*	0,78	0,41	0,42	0,95	0,41	***2*,*02***
*lower-middle*	0,72	0,22	0,26	0,89	0,28	***2*,*63***
*low*	0,67	0,05	0,09	0,70	0,09	***3*,*40***

Independent of the income class, countries score best in the Mobile Devices category, closely followed by the average Internet Speed Factor. The biggest discrepancies exist with the Hardware Factor, which is closely linked to the Internet-Users Factor: people with PCs using them to access the Internet. The Education Factor has significant differences among high and low income countries; the lowest scores are also found in the Educational Factor: this is also where high-income countries have most potential to improve. Further improvements in the overall DPI score for many developing countries are likely to happen with the increased use of PCs and hence the probable increased access to the Internet and its digital resources (e.g., data, software, training).

Although there are gaps in the datasets obtained from the World Bank and United Nations, the results of this study indicate the potential of the DPI to monitor the sharing of digital data and information. Unlike the World Risk Index (http://www.worldriskreport.com, 2014), all the datasets used with the Data Poverty Index are currently freely available, with annual updates (Table 5). Moreover, the important Internet Speed factor is relatively independent of potential government interference [32]. Hence, it should be possible to monitor annual changes in access to digital information via the DPI, which would make it a potential metric for monitoring the Sustainable Development Goals [33]. The DPI could be combined with World Bank’s *‘Index of Risk Preparation Across Countries’* (IRPAC) [34]. Unfortunately the IRPAC is not downloadable for individual countries and the World Bank is vague about the variables involved in the IRPAC calculation.

**Table 5:** Comparison of input variables of global indices dealing with global disaster risk.

**Index**	Used Indicators
**Data Poverty Index**	
Values available online; factors un-weighted.	Mobile cellular subscriptions per 100 people; Country population;
	Telephone quality: % of population covered by mobile cellular network;
	Individuals using Internet, % of population; Tertiary Gross enrolment ratio;
	% households with a computer; Number of universities in a country;
	Internet upload speed (qualifying date: 10.12.2013);
	Internet download speed (qualifying date: 10.12.2013).
**ICT Development Index (2012)** [39]	
Values only available in report; factors weighted.	*Adult literacy rate* *^1^; Mobile telephone subscriptions per 100 people;
	% households with a computer; Fixed-telephone lines per 100 inhabitants;
	Fixed (wired)-broadband Internet subscriptions per 100 inhabitants;
	Secondary gross enrolment ratio; Tertiary gross enrolment ratio;
	**International Internet bandwidth (bit/s) per Internet user;**
	**Active mobile-broadband subscriptions per 100 inhabitants.**
**World Bank: Index of Risk Preparation Across Countries (IRPAC)** [34]	
Values not available; factor weighting not stated.	% of population with access to improved sanitation facilities.
	Immunization rate for measles; **Indicator of fiscal space based on gross public debt as a % of revenues (state support);**
	**Average years of total schooling for the population aged 15 or over;**
	**Proportion of households with less than $1,000 in net assets;**
	**% of work-force who contribute to a pension scheme;**
	**Proportion of respondents stating that “in general, people can be trusted” (social support); Index of access to finance.**
**UN World Risk Index (2014)** [40]	
Values available in report and online; factors weighted by expert knowledge.	Total population of country; Number of physicians per 10,000 people;
	Share of the population without access to improved sanitation;
	Share of the population without access to an improved water source;
	Number of hospital beds per 10,000 people; Adult literacy rate;
	Gross domestic product per capita (purchasing power parity);
	Public health expenditure; Private health expenditure; Gini index;
	Dependency ratio (share of under 15- and over 65-year-olds in relation to the working population); Combined gross school enrolment;
	*Extreme poverty population living with USD 1*.*25 per day or less* *^2^;
	*Number of people in a country who are exposed to (A) earthquakes*, *(B) cyclones and/or (C) flooding* *^2^; *Good governance (Failed States Index)* *^3^;
	*Number of people in the country threatened by(D) drought and/or (E) sea level rise (each half-weighted owing to database uncertainty)* *^2^;
	*Share of female representatives in the National Parliament* *^3^;
	*Life expectancy at birth* *^3^ *; Biodiversity and habitat protection* *^3^;
	*Share of female representatives in the National Parliament* *^3^;
	*Water resources* *^3^; *Corruption Perceptions Index* *^3^; *Forest management* *^3^; *Agricultural management* *^3^;
	**Insurance (life insurance excluded).**

Legend for the Used Indicators column: Data sets freely available, apart from *Italics*:

*^1^ dataset freely available but patchy and inconsistent coverage of countries;

*^2:^ data not up-to-date, last updated in 2007 or 2008;

*^3:^ data is not up-to-date, last updated in 2010.

Bold: data not freely available.

A number of indicators collected by the ITU would be of interest for evaluating a technology-related data poverty index. However, many of the ITU data sets are not freely available and hence they were not considered in this study, since only freely available data allows the construction of a sustainable and verifiable index. Listed below are ITU World Telecommunication Indicators that are of potential use but are currently not freely available to further improve the DPI:

- Mobile cellular network:
  - Percentage of the population covered by at least a 3G mobile network
- Fixed Internet:
  - Fixed (wired) Internet subscriptions
  - Fixed (wired)-broadband 2 Mbit/s to less than 10 Mbit/s subscriptions
- Wireless broadband:
  - Active mobile-broadband subscriptions
  - Dedicated mobile-broadband subscriptions
- Tariffs:
  - Mobile-cellular monthly subscription charge, in US Dollars (USD)
  - Mobile-cellular prepaid connection charge, in USD
- Investment:
  - Annual investment in mobile communication services, in USD
- Household ICT access and individual use:
  - Percentage of households with computer
  - Percentage of individuals using a computer
  - Percentage of households with Internet
  - Percentage of individuals using the Internet
  - Percentage of households with mobile-cellular telephone
  - Percentage of individuals using a mobile cellular telephone

(source: http://www.itu.int/en/ITU-D/Statistics/Pages/stat/default.aspx)

## Discussion

The index that we have presented here is the first to quantify and visualise the problem of global data poverty. Fig 3 shows that the level of data poverty does not necessarily correspond to the income classification of the World Bank. For instance, Italy, Antigua and Barbuda, Oman, Trinidad and Tobago are among the World Bank’s high income countries, but they do not attain the top score of minimal data poverty category. For the first three it is mainly due to their slow download speeds, relative to other high-income nations. Trinidad and Tobago, as well as Antigua and Barbuda, gained only moderate scores because of their relatively low score in the enrolment in tertiary education. On the other hand, Belarus, Bulgaria, Hungary, Kazakhstan, Lebanon and the Republic of Macedonia scored better than one might expect for data poverty, if only considering their income class. At the other end of the scale are Burkina Faso and Myanmar, with low scores in all categories, while Tajikistan (a low-income country) is close to the ‘below-average’ level of data-poverty, due to the high scores in Internet speed and the variables linked to mobile devices and networks.

In some countries, particularly in Africa, mobile phone usage is more widespread than Internet usage, which should be taken into account when developing VGI applications or preparing geoinformatic training materials. Experience suggests that Internet access and usage in developing countries primarily occurs in cities. The Data-Poverty Index is thus able to give an indication of societal structure and discrepancies in urban versus rural data resources. This needs to be investigated further, with more detailed data than national averages. In most countries, the data used to compile the Data Poverty Index should be available down to district levels of administration. For a given district or city, local authorities or volunteers could add data about Internet speed by local online testing. Maps of Internet-speeds and mobile network coverage could then be produced, enabling analysis of the variations between districts or cities [31]. The technological capabilities for disaster preparedness, response and recovery could be examined at district level (e.g. How difficult is it to get freely available, internet-deliverable satellite imagery for different districts of a given country via Internet downloads? Is it possible to use applications with mobile devices and crowd-source mapping in all of the districts of a given country?) Districts or cities with data poverty issues could be detected and targeted for improvements, thus assisting development activities and disaster risk reduction.

Data poverty topics for further consideration include differences between urban and rural areas and their corresponding mobile phone network or Internet availability, as well as differences between relatively flat or mountainous regions, all of which could not be examined with the available national datasets. This national-scale data limitation might change with the plans of Google and Facebook to expand Internet availability, particularly in developing countries, using drones, balloons or small satellites [35].

In many developing countries access to the Internet is more limited than mobile phone network coverage. Particularly in Africa, mobile phones are being used to provide digital services that greatly benefit stakeholders, such as micro-banking or weather forecasts for farmers [36–38]. The need to include a metric for Internet access—independent of whether it is via Desktop PCs, laptops or mobile devices—is reinforced by the increased use of social media, which gives its users access to early warnings, as well as providing numerous potential ‘human sensors’ for monitoring crisis events and assisting with disaster response [23–25]. On a cautionary note, too much dependence on information technologies could increase disaster risk, should there be a failure in the tele-communications system. “Reliance on mobile phones or the Internet to issue disaster warnings or make financial transactions, may reduce resilience where power supplies are exposed to hazards” [37].

The DPI has some similarities to the ICT Development Index (IDI) presented in 2012 by the International Telecommunications Union. However, the IDI was linked with GDP to examine economic developments, rather than looking at implications for disaster risk reduction. Further differences with the DPI are that the IDI does not distinguish between upload and download speed in its evaluation and it also uses a different education factor [39]. Information technologies have not been considered in any of the UN World Risk Reports since 2011. The World Risk Reports consider education, but only with respect to the literacy rate; while the DPI looks at information technology literacy and university education (Table 5).

In summary: severe data poverty results in limited development, with high vulnerability and poor resilience to the impacts of hazards. With the financial pressures and limited human resources experienced by most of the world, the development and application of free geoinformatics is a significant step towards sustainable development [18]. Disaster response and mitigation will be more difficult in countries with severe data poverty, due to limited digital infrastructure and few specialists in information technologies. The DPI highlights countries where support is needed for improving access to the Internet and for the provision of training in geoinfomatics: that should facilitate increased use of geospatial data and GIS-generated maps by the planners, political decision-makers and emergency managers of those countries. With regard to its application, the DPI uses freely available data and provides a rapid method for annually monitoring the provision of digital data and information, on a country by country basis: it is thus of potential use for monitoring Sustainable Development Goals of the Sendai Framework for Disaster Risk Reduction [15].
