# Correction: Quantifying and Mapping Global Data Poverty

**DOI:** 10.1371/journal.pone.0145591

**Published:** 2015-12-17

**Authors:** Mathias Leidig, Richard M. Teeuw

There is an error in the caption of Table 3. Please view the corrected Table 3 here.

**Table 3:** Example scores of the Data Poverty Index (DPI) and relationships to the World Bank income classification.

Top Scores		Bottom Scores	
*Country*	*Score (max*. *5)*	*Country*	*Score (max*. *5)*
1. Iceland	0.17	.. 83. China*	2.05
2. Norway	0.36	.. 109. Indonesia	2.75
3. Finland	0.39	.. 114. Nigeria	2.88
4. Estonia	0.51	.. 129. India	3.16
5. Denmark	0.52	.. 142. Benin	3.49
.. 8. U.S.A	0.55	148. Congo, Dem.	3.67
.. 17. United Kingdom	0.71	149. Malawi	3.72
.. 21. Germany	0.76	150. Yemen	3.78
.. 23. Japan	0.77	151. Myanmar	3.95
.. 39. Russia	1.04	152. Burkina Faso	4.04
**2014 World Bank income classification**		**Data Poverty Index Range**	
Low-income countries		4.04–2.62	
Lower-middle income countries		3.78–1.41	
Upper-middle income countries		3.32–0.97	
High-income countries		1.53–0.17	

Scores: < 1.21, low data poverty; 1.21–2.42, below average data poverty; 2.42–3.62, above average data poverty; > 3.62, high data poverty. Remark: Only countries with a complete dataset have been considered.

* China Mainland, excluding Macao and Hong Kong.
